# Talonavicular Osteochondral Lesions: Surgical Technique and Clinical Outcomes from the Boston and Amsterdam Perspectives

**DOI:** 10.1177/19476035231200334

**Published:** 2023-09-26

**Authors:** Quinten G.H. Rikken, Jari Dahmen, Arianna L. Gianakos, Lorena Bejarano-Pineda, Gregory Waryasz, Christopher W. DiGiovanni, Sjoerd A.S. Stufkens, Gino M.M.J. Kerkhoffs

**Affiliations:** 1 Department of Orthopedic Surgery, Amsterdam Movement Sciences, Amsterdam UMC, Location AMC, University of Amsterdam, Amsterdam, The Netherlands; 2 Academic Center for Evidence-Based Sports Medicine (ACES), Amsterdam UMC, Amsterdam, The Netherlands; 3 Amsterdam Collaboration on Health & Safety in Sports (ACHSS), International Olympic Committee (IOC) Research Center, Amsterdam UMC, Amsterdam, The Netherlands; 4 Foot & Ankle Research and Innovation Lab, Massachusetts General Hospital, Harvard Medical School, Boston, MA, USA

**Keywords:** articular cartilage, tissue, OCL, talonavicular joint, surgical

## Abstract

**Purpose:**

The primary purpose of the present study was to assess the patient-reported outcomes, complications, and reoperation rate of patient who underwent surgical treatment for symptomatic osteochondral lesions of the talonavicular joint (TNJ).

**Methods:**

Patients undergoing surgical treatment for symptomatic osteochondral lesions of the TNJ with a minimum of 12-month follow-up were included. Outcomes included clinical patient-reported outcome measures (PROMs), return to sports and work outcomes, and postoperative complications or reoperations. Medical records were screened by 2 independent reviewers. Patients were contacted by phone and underwent an in-depth interview. Additionally, operative techniques for both arthroscopic and open surgical approaches for treating TNJ osteochondral lesions were described.

**Design:**

Retrospective Case Series (Level IV) and Surgical Technique.

**Results:**

A total of 7 patients were included with a final follow-up time of 25.4 (SD: 15.2) months follow-up. PROMs were considered satisfactory for 5 out of 7 patients, 6 out of 7 patients returned to any level of sports at a mean of 3.7 (SD: 4.2) months, and 5 out of 6 patients returned to preinjury level of sports at a mean of 14 (SD: 7.5) months. All patients returned to work at an average of 5.4 (SD: 3.6) weeks. No complications or reoperations after index surgery were reported.

**Conclusion:**

Surgical treatment of TNJ osteochondral lesions is a feasible procedure that may offer successful clinical, sport, and work outcomes in the majority of patients. Both open and arthroscopic surgical treatments are available and can be considered in a patient-specific treatment plan.

## Introduction

The majority of osteochondral lesions in the foot and ankle are primarily located on the talar dome; different locations such the talonavicular joint (TNJ) have been described causing important function disability and chronic pain.^1^ Even though these lesions may be rare, patients may present with mid-foot pain during or after weightbearing, which limits their participation in sports or physical activities including work-related activities. In turn, this lesion may be the starting point for the development of TNJ osteoarthritis which may require joint fusion, which can be severely debilitating. Therefore, it is paramount to adequately treat TNJ osteochondral lesions to regain function and reduce pain for patients as well as to prevent joint degeneration.

Osteochondral lesions of the TNJ have been described in only a handful of case reports and case series studies.^2-11^ Likewise, clinical and sport-related outcomes have been reported in less than 15 patients.^2,4,7,9-11^ The evidence-based treatment of these lesions is therefore limited to expert opinion. Improving the understanding of the outcomes following treatment for osteochondral lesions of the TNJ might improve treatment strategies which would be helpful for clinicians in a patient-specific treatment algorithm.

The primary aim of this study was to assess the patient-reported outcomes (including clinical, sport, and work-related outcomes), complications, and reoperations in a selected cohort of patients who underwent surgical for an osteochondral lesion of the TNJ. The secondary aim of this article was to describe the surgical technique for both open and arthroscopic approaches for the treatment of TNJ osteochondral lesions from the perspective of 2 expert centers.

## Methods

This study included a single-institution cross-sectional follow-up of surgically treated osteochondral lesions of the TNJ at the department of Orthopeadic Surgery and Sports Medicine of Amsterdam UMC, The Netherlands, and an in-depth description of both arthroscopic and open approaches from the perspective of 2 expert centers in the treatment of cartilage injuries in the foot and ankle, namely the department of Orthopeadic Surgery and Sports Medicine of Amsterdam UMC, The Netherlands and the Foot & Ankle Service of Massachusetts General Hospital, Boston, USA. Approval by the local medical ethics committee at the department of Orthopeadic Surgery and Sports Medicine of Amsterdam UMC, The Netherlands, was obtained prior to the start of this study (reference number: MEC 08/326) and the study was performed in accordance with the Declaration of Helsinki.

### Patient Selection

Surgically treated patients for a primary or non-primary (i.e., failed prior surgical treatment) osteochondral lesion of the TNJ between January 2015 and August 2022 were eligible for inclusion in this cross-sectional follow-up study. Patients underwent a minimum of 3 to 6 months conservative therapy, and if unsuccessful, surgical treatment was considered. Conservative therapy consisted of, or a combination of, moderation of physical activities, physical therapy, bracing, inlays, and non-steroid anti-inflammatory medication. The operative records from 2 senior foot and ankle fellowship-trained surgeons (G.K. and S.A.S.) were screened in order to identify potential eligible patients. The exclusion criteria are listed in **Table 1**.

**Table 1.** Exclusion Criteria for Patient Selection.

End-stage osteoarthritis of the TNJ (i.e., complete joint destruction with no joint line recognizable on imaging)
Polytrauma patients, including open fractures of the talonavicular joint
Follow-up <12 months
Patients lost to follow-up or unwilling to participate

TNJ = talonavicular joint.

### Data Collection

Eligible patients were contacted by phone after identification in order to obtain consent for participation in the study and an interview. After obtaining informed consent, patients were sent an online questionnaire, which was distributed via the CASTOR© portal, in order to collect patient-reported outcome measures (PROMs). Subsequently, patients underwent an in-depth interview by phone regarding any surgical treatment for the lower extremities after the index surgery, return to sports (RTS), and return to work.

Patient demographics and characteristics of the osteochondral lesion and its treatment were extracted from the medical electronic records. Patient demographics and injury characteristics included sex, age, body mass index (BMI), participation in sports and at what level (i.e., none, amateur, competitive, or professional), injury circumstances (i.e., traumatic—fracture or sprain—or sudden onset), and concomitant injuries. Lesion characteristics included the primary or secondary (i.e., failed primary surgical treatment) lesion type as well as a radiological assessment. Treatment characteristics included open or arthroscopic approach, concomitant procedures during the surgical intervention, and any previous surgical treatment for the foot and ankle.

### Clinical Outcomes

The primary outcome of this study was defined as the Numeric Rating Scale (NRS) during weightbearing at final follow-up. The NRS is a subjective pain scale from 0 (no pain) to 10 (worst pain imaginable).^12^ A NRS during weightbearing <4 was considered as a successful treatment outcome.

Additional reported clinical outcomes included the NRS at rest, the Foot and Ankle Outcome Score (FAOS), and the American Orthopaedic Foot and Ankle Society Ankle-Hindfoot score (AOFAS).^13,14^ The AOFAS is a 100-point, physician-administered, clinical outcome scale. Its subcategories consist of pain (40 points), function (50 points), and alignment (10 points). The FAOS is a PROM consisting of 42 questions distributed over 5 subscales: symptoms, pain, activities of daily living, sport, and quality of life.

### Return to Sports and Work

Return to sport and work information were collected using the patient electronic records, CASTOR© portal, and verified by in-depth telephone interview. Patients were asked about the participation in any sport and level of sport (recreational, competitive, or professional level) preinjury, as well as the hours per week participating in sport preinjury and postoperatively. Return to sports was defined according to Ardern *et al.*,^15^ which includes return to any level of sports, return to preinjury level of sports, and return to performance (higher level of sports than preinjury). Additionally, the time for each eligible subcategory of return to sports was collected.

### Radiological Assessment

**Figure 1.**
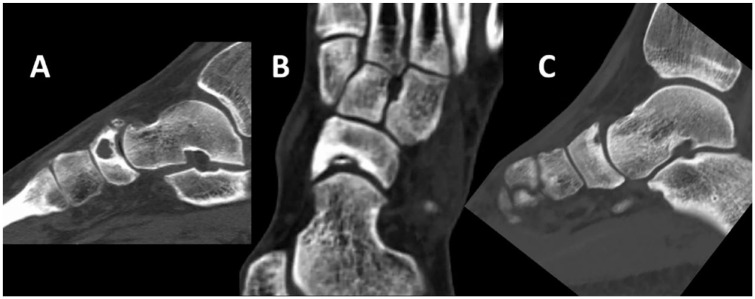
Examples of morphological descriptions: (**A**) cystic lesion, (**B**) fragment, and (**C**), crater.

In order to assess preoperative lesion characteristics, an assessment of preoperative computed tomography (CT) scans was performed by 2 independent reviewers. Lesion characteristics included the lesion size in millimeters (mm) from anterior-posterior (AP), medial-lateral (ML), and depth. The lesion morphology was assessed for the presence of cysts, crater, or fragments (see **Fig. 1**). Additionally, lesion location (i.e., medial, central, or lateral) and localization (i.e., talar-sided or navicular-sided lesion) were assessed.

### Complications and Reoperations

Any complications related to the surgery or reoperations of the foot/ankle were extracted from the medical records and were verified during the telephone interview. Revision surgery was defined as any surgical procedure involving treatment of the cartilage of the TNJ after the index procedure.

### Surgical Techniques

The present study describes both an arthroscopic technique and open technique in order to treat osteochondral lesions of the TNJ. Prior to surgical treatment, patients receive a combined popliteal and saphenous nerve block performed by the anesthesia service followed by induction with general anesthesia. The operative field is pre-scrubbed with chlorhexidine and then prepped in a sterile fashion. Both procedures begin with the application of an Esmarch bandage and insufflation of the lower extremity tourniquet.

#### Arthroscopic debridement with cartilage extracellular matrix and platelet-rich plasma application: The Boston perspective

The TNJ is localized using mini C-arm fluoroscopy. Two portals can be utilized, including a 1-cm incision medial to the tibialis anterior tendon, directly over the TNJ, and a slightly less than 1 cm dorsal to the medial incision and medial to the extensor hallucis longus tendon as previously described by Ross *et al.*^9^

**Figure 2.**
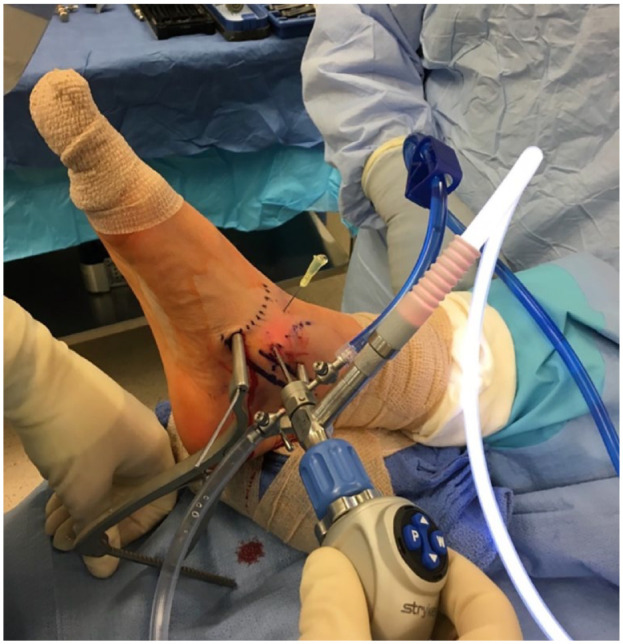
Two portals can be utilized including a 1-cm incision medial to the tibialis anterior tendon, directly over the talonavicular joint and a slightly less than 1 cm dorsal to the medial incision and medial to the extensor hallucis longus tendon as previously described by Ross *et al.*

Open dissection is performed between the interval of the tibialis anterior and the extensor hallucis longus tendons. The periosteum overlying the TNJ is then dissected off, exposing the TNJ longitudinally. Direct inspection of the joint is performed. A Hintermann retractor is placed between the navicular and talus to distract the joint allowing better access to the osteochondral lesion, see **Figure 2**. In certain cases, a small arthroscope (1.9-mm scope) may be utilized to better assess the joint surface particularly with more plantarly located lesions. Needle arthroscopy has become an attractive alternative as a minimally invasive approach when evaluating small joints and can be used at the surgeon’s discretion.^16,17^

**Figure 3.**
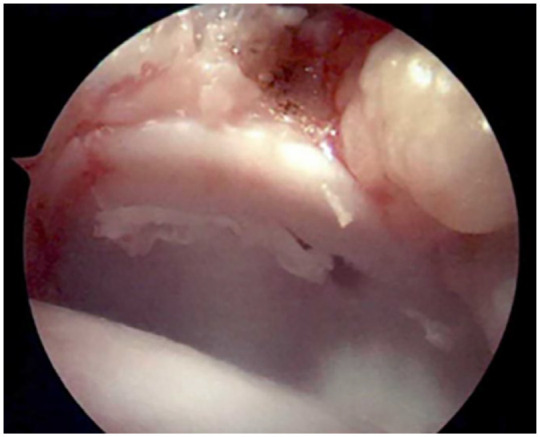
Arthroscopic image demonstrated the osteochondral lesion.

Any loose cartilage fragments or fraying around the rim of the defect (see **Fig. 3**) are debrided using a scalpel and curettes. After final debridement, the osteochondral lesion is measured and microfracture drilling is performed using a 0.6- to 1.5-mm K-wire or microfracture awl.

**Figure 4.**
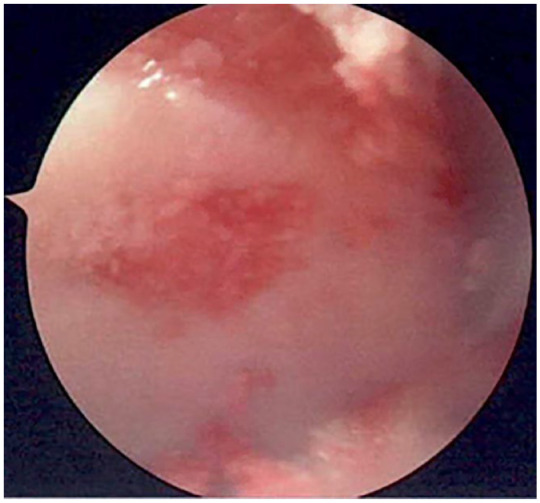
Arthroscopic image demonstrating the result of the procedure after the cartilage matrix has been placed into the lesion.

There have been a variety of techniques described with regard to the type of biologic grout utilized when filling the osteochondral defect. The lesion may be filled using a cartilage extracellular matrix mixed with platelet-rich plasma (PRP). This matrix contains type II collagen and proteoglycans to serve as a scaffold over the defect.^18^ First, a thin layer of fibrin glue is applied over the defect. The mix is then placed and smoothens within the defect. Verify the mix is flushed to the cartilage surface and avoid “overstuffing” the defect to prevent a prominent construct. A final layer of the fibrin glue is applied over the entire defect, this can also be visualized arthroscopically, after which the construct should not be manipulated for 5 minutes, see **Figure 4**.

Once the fibrin glue is dried, the Hintermann retractor is removed, and the capsule is closed using absorbable suture. Incision is copiously irrigated and closed by layers. A final visualization of the defect is performed under fluoroscopy and the remaining PRP is injected into the TNJ. A short leg well-padded splint is applied.

Postoperatively, the sutures are removed, and the patient is transitioned into a tall aircast walking boot with the use of crutches at 2 weeks. The patient will remain non-weightbearing for the first 2 weeks. Physical therapy usually starts at week 2 for ankle plantarflexion and dorsiflexion. Progressive weightbearing is started gradually over the second through sixth weeks and patient will begin eversion and inversion range of motion. Patient is weaned out of the boot between weeks 8 and 10. Return to sports typically occurs between 3 and 6 months after surgery when the patient has achieved sport-specific strength, stability, and function.

Clinical pearls:

- For plantar lesions, it is recommended using an arthroscope or needle arthroscope to have a better visualization even if the surgery is performed open.
- A Hintermann distractor may be useful for increasing the joint space allowing better visualization.
- Ensure the fibrin glue is dried prior to letting down traction.
- The cartilage extracellular matrix can be mixed with bone marrow aspirate concentrate (BMAC) instead of PRP.

Pitfalls:

- Make sure there is not a navicular stress fracture or other associated injury that needs to be addressed within the same procedure.

#### Open debridement and bone marrow stimulation with or without autologous cancellous bone grafting from the iliac crest: The Amsterdam perspective

After careful identification of the TNJ under fluoroscopy, a dorsal incision is made over the TNJ. Hereafter, the saphenous nerve and vein are identified and protected such that open dissection unto the periosteum of the TNJ can be performed. The periosteum overlying the TNJ is then dissected, exposing the TNJ longitudinally. Similar to the arthroscopic technique, a Hintermann spreader is applied over the talus and the navicular bone in order to improve access to the TNJ. Direct inspection of the joint and identification of the osteochondral lesion is performed, also comparing these to size and morphology on the preoperative CT scan. After identification, the defective articular cartilage of the osteochondral lesion is debrided and the sclerotic subchondral bone curetted until healthy bone is observed. Hereafter, the subchondral bone is drilled multiple times using a 2.5-mm drill. After observing bleeding of the drilled lesions site, the TNJ is irrigated and closed in layers.

In case the lesion size (diameter and/or depth) is too large (i.e., >1/3 of joint surface area or massive cyst) for debridement and bone marrow stimulation (BMS) alone, or in case of large cysts, it can be chosen to fill the lesion site after drilling with an autologous cancellous bone graft from the iliac crest. This decision is surgeon and lesion specific and, therefore, no absolute lesions size or cyst size guidelines warranting the use of this technique can be provided. Harvesting of the autologous bone graft is started by identification of the ipsilateral iliac crest. In the next step, open dissection unto the bone is performed. Access to the iliac crest is created using a small chisel whereafter, by means of a curette, cancellous bone graft tissue is collected. Hereafter, the lesion site is packed with the harvested cancellous bone. After achieving adequate stable compression of the cancellous bone in the lesion site, the TNJ and iliac crest are diligently irrigated and closed in layers. After both procedures, a short leg splint is applied which is changed to a pressure bandage 1 day postoperatively. When swelling has reduced, and the sutures have been removed 2 weeks postoperatively the patient can start passive and active non-weightbearing range of motion and strengthening exercises with the help of an experienced physical therapist. Patients are kept non-weightbearing up to 6 weeks postoperatively. Hereafter, a personalized rehabilitation program focused on regaining normal gait, balance, and strength of the ankle is started.

Clinical pearls:

- Compare intraoperative osteochondral lesion morphology and size to preoperative imaging (MRI or CT scan).
- Perform adequate debridement and drilling of the sclerotic bone until bleeding.

Pitfalls:

- Inadequate access to the TNJ by not using a Hintermann spreader.

### Patient Selection and Demographics

In total, 8 patients were eligible for inclusion. Seven patients were contacted and included in the present study, 1 patient could not be contacted and was lost to follow-up. All patients failed conservative therapy. Of the 7 patients, 6 were female and 1 was male, with a mean BMI of 22.4 kg/m^2^ (SD: 2.8). At the time of surgery, mean age was 25.1 (SD: 13.9) years old. The mean follow-up time was 25.4 (SD: 15.2) months. **Table 2** provides a full overview of demographic and treatment characteristics at baseline for each patient.

**Table 2.** Overview of Baseline Patient, Lesion, and Treatment Characteristics for Each Patient.

Case	Patient Characteristics					Lesion Characteristics			Treatment Characteristics			
	Sex	Age at Surgery, Years	BMI, kg/m^2^	Preinjury Sports	Injury Etiology, Trauma	Lesion Location	Lesion Size, mm	Morphology	Previous Surgical Treatment	Surgical Treatment	Additional Procedures	FU Time, Months
1	F	17	18.6	Football, competitive level	No, sudden onset	Navicular, medial	AP: 15 ML: 13 Depth: 4	Fragment	None	Open debridement and BMS	None	46
2	F	20	23.6	Track & Field, professional level	No, sudden onset	Navicular, central	AP: 7 ML: 3 Depth: 2	Fragment	None	Open debridement and BMS	None	39
3	M	20	24.0	Track & Field, professional level	No, sudden onset	Navicular, central	AP: 5 ML: 8 Depth: 4	Crater	None	Open debridement and BMS	None	39
4	F	44	23.0	Dancing, recreational level	No, sudden onset	Navicular, lateral	AP: 10 ML: 18 Depth: 9	Cyst	None	Open autograft transplantation	None	18
5	F	46	26.4	Cycling, recreational level	No, sudden onset	Navicular, lateral	AP: 14 ML: 16 Depth: 8	Cyst	None	Open autograft transplantation	None	12
6	F	11	18.7	Gymnastics, recreational level	Yes, repetitive inversion spraining	Talus, central	AP: 9 ML 9 Depth: 6	Crater	None	Open debridement and BMS	None	12
7	F	18	22.6	Dancing, recreational level	Yes, repetitive inversion spraining	Navicular, central	AP: 7 ML: 6 Depth: 4	Crater	None	Open debridement and BMS	None	12

BMI = body mass index; FU = follow-up; AP = anterior-posterior; ML = medial-lateral; BMS = bone marrow stimulation.

### Statistical Analysis

Data were analyzed using Stata 15 (StataCorp LP, College Station, TX). Continuous variables are reported per patient and as means with standard deviations. Dichotomous variables are reported per patient and as numbers with percentages. Due to the low number of patients involved in the present case series, no formal statistical comparison of outcomes was made. Radiological measurements were performed by 2 independent assessors and continuous outcomes are reported as the means from both measurers.

## Results

### Clinical Outcomes

The primary outcome, the mean NRS during weightbearing at follow-up, was 2.1 (SD: 2.7) out of 10. Five patients achieved a successful treatment outcome at final follow-up. One (case no. 1) of the 2 patients with an unsuccessful treatment outcome had the same procedure (open debridement and BMS) on the contralateral ankle at another hospital 23 months after the index procedure. **Table 3** provides an overview of clinical outcomes for each patient.

**Table 3.** Overview of Clinical Outcomes at Final Follow-Up per Patient.

Case	FU Months	Treatment	NRS			FAOS					AOFAS
			Rest	WB	Running	Symptoms	Pain	ADL	Sport	QoL	
1	46	Open debridement and BMS	3	6	6	39	69	100	50	38	88
2	39	Open debridement and BMS	1	0	0	64	92	100	100	88	100
3	39	Open debridement and BMS	1	1	1	79	92	100	70	69	100
4	12	Open autograft transplantation	0	0	3	96	100	100	85	88	100
5	12	Open autograft transplantation	0	2	2	79	92	100	65	56	90
6	12	Open debridement and BMS	0	0	1	96	94	100	90	75	90
7	12	Open debridement and BMS	3	6	6	68	50	97	65	44	77
Total	25.4 (SD: 15.2)		1.1 (SD: 1.3)	2.1 (SD: 2.7)	2.7 (SD: 2.4)	74.4 (SD: 19.9)	84.1 (SD: 17.9)	99.6 (SD: 1.1)	75.0 (SD: 17.3)	65.4 (SD: 20.1)	92.1 (SD: 8.6)

FU = follow-up; NRS = Numeric Rating Scale; WB = weightbearing; FAOS = Foot and Ankle Outcome Score; ADL = activities of daily living; QoL = quality of life; AOFAS = American Orthopaedic Foot and Ankle Society Ankle-Hindfoot score; BMS = bone marrow stimulation.

### Return to Sports and Work

Preoperatively, 2 patients participated at a professional level of sports, 2 patients participated in competitive level of sports, and 3 patients participated in recreational level of sports (**Table 4**). At final follow-up, 6 patients returned to any level of sports at a mean of 3.7 (SD: 4.2) months, 4 patients returned to preinjury level of sports at a mean of 14 (SD: 7.5) months, and 2 patients returned to performance at a mean of 32 months. One patient (case no. 7) did not return to sport due to residual pain complaints. An overview of the return-to-sport outcomes per patient is provided in **Table 4**.

**Table 4.** Overview Return-to-Sport Outcomes.

Case	Preinjury Sports and Level	Hours per Week	Follow-Up Sports and Level	Hours per Week	RTS Any Level	RTS Any Level Time	RTS Preinjury Level	RTS Preinjury Level Time	Return to Performance	Return to Performance Time
1	Football, competitive level	8 hours	Football, competitive level	3 hours	Yes	12 months	No	N/A	No	N/A
2	Track & Field, professional level	20 hours	Track & Field, professional level	24 hours	Yes	1 month	Yes	24 months	Yes	33 months
3	Track & Field, professional level	6 hours (due to progressive complaints)	Track & Field, professional level	15 hours	Yes	1 month	Yes	4 months	Yes	31 months
4	Dancing/Running, both recreational level	Both 2 hours	Dancing/Recreational level, both recreational level	Both 2 hours	Yes	2 months	Yes	18 months	No	N/A
5	Cycling, recreational level	8 hours	Cycling, recreational level	8 hours	Yes	4 months	Yes	12 months	No	N/A
6	Tennis, recreational level	2 hours	Tennis, competitive level	4 hours	Yes	2 months	Yes	12 months	No	N/A
7	Dancing, recreational level	1 hours	None	N/A	No	N/A	No	N/A	No	N/A

RTS = return to sport; N/A = not applicable.

With regard to the return-to-work outcomes, all patients who worked preoperatively returned to their preoperative occupation. The mean return to work time was 5.4 (SD: 3.6) weeks. An overview of the return-to-work outcomes for each patient is available in **Table 5**.

**Table 5.** Overview Return-to-Work Outcomes.

Case	Preinjury Occupation	Work Hours per Week	Return-to-Work Time	Follow-Up Occupation	Work Hours per Week
1	Student (high-school) Retail (sitting)	32 24	8 weeks	Education (sitting)	32
2	Student (high-school)	32	3 weeks	Office Job (sitting)	20 (part-time due to sports)
3	Healthcare (standing)	12	3 weeks	Healthcare	12
4	Healthcare (sitting)	28	12 weeks	Healthcare (sitting)	28
5	Office Job (sitting)	32	2 weeks	Office Job (sitting)	32
6	Student (primary school)	32	6 weeks	Student (high-school)	32
7	Student (high-school)	32	4 weeks	Student (high-school) Sales Job (standing—part-time)	32 12

### Radiological Examination

Radiological assessment by means of baseline CT scan revealed that all but one lesion were navicular sided, with 4 lesions located centrally, 2 lesions located laterally, and 1 lesion located medially. The mean lesion size was 9.6 (SD: 3.7) mm in AP and 10.4 (SD: 5.4) mm in ML directions, and 5.3 (SD: 2.5) mm depth. Lesion morphology was a crater in 3 cases, cystic in 2 cases, and fragment in 2 cases. An overview of the radiological characteristics is available in **Table 2**.

### Complications and Reoperations

No major complications were noted in any of the included patients, nor were any complications noted at the harvest site for patients undergoing cancellous bone grafting for the iliac crest. One patient (case no. 7) developed soft-tissue impingement at the TNJ and a sinus tarsi syndrome due to neuromuscular deficits, which at the time of writing is being treated with physical therapy. No reoperations or revision surgery of the affected foot and ankle after the index surgery were reported.

## Discussion

The main finding of this study is that surgical treatment for osteochondral lesions of the TNJ may lead to successful outcomes in the majority of patients. Additionally, most patients were able to return to sports, and all professional athletes were able to return to a performance level. The present article includes an in-depth description of the surgical techniques from 2 expert centers in the treatment of foot and ankle cartilage pathologies based in the United States and Europe. The findings of this case series study may be of benefit to clinicians which encounter these lesions and could help inform their patients.

Osteochondral lesions of the TNJ are an interesting, though challenging entity for clinicians. Their incidence is reported as rare, and only a handful of case studies and case series are available in the literature.^2,5,7,9-11^ It is thought that both a traumatic and non-traumatic origin may play a role in their development.^4,6,9,10,19^ Interestingly, TNJ osteochondral lesions have been reported to coexist or develop after a (high-grade) TNJ stress fracture.^4,6,7,10^ It has been hypothesized that this is due to the poor blood supply of the navicular bone, resulting in a “watershed” area which may be more susceptible to injury and carries poor healing characteristics.^4^ Moreover, one could also hypothesize that TNJ osteochondral lesions are more commonly present asymptomatically and that a period of avascularity or poor blood supply during skeletal development may yield a susceptibility to these lesions after trauma, a theory previously proposed in bilateral talar osteochondral lesions.^20^

### Clinical and Sport Outcomes

There is paucity in the current literature regarding the clinical and sport outcomes of patients with talonavicular osteochondral lesions.^2,5,7,9-11^ These reports include 2 case series both with reported outcomes in 3 patients.^9,10^ Saxena and Fullem^10^ showed the outcomes of open debridement and bone marrow stimulation with concomitant arthrodiastasis. Among these 3 patients were 2 patients with concomitant navicular stress fractures. One of the 3 patients participated in professional soccer. All 3 patients returned to sports and, from the available data, 2 out of 3 returned to preinjury level of sports, including the professional athlete. Ross *et al.*^9^ reported on patient-reported outcomes and sport outcome following arthroscopic debridement and BMS. The authors found an improvement in the functional FAOS for all 3 patients as well as an improvement in the quality of life short-form 12 (SF-12) questionnaire. The authors found that on 1-year postoperative magnetic resonance imaging (MRI), a stable fibrocartilage had formed and the bone edema was resolved. In terms of sport outcomes, the authors found that all 3 patients returned to their preinjury level of sports or activities. When considering the outcomes reported in the present study, it can be stated that both for the PROMs and return-to-sport outcomes by and large concur with the sparse body of clinical literature reported to date.^2,4,7,9-11^ In terms of treatment strategies, the majority of the patients included in this study underwent debridement and BMS, as is common in the literature.^2,7,9-11^ However, we also treated 2 cases with an autograft transplantation with good results, which has also been described with favorable results by Kanazawa *et al.*^6^ Both treatment options seem possible and safe, and can considered as possible surgical options according to our current understanding of their outcomes. It was not possible to compare both techniques as the number of included patients did not allow for reliable statistical comparisons. The addition of biological adjuncts, such as BMAC, may prove an interesting opportunity for future research as previous studies have shown that there may be an added benefit in cartilage surgery,^21^ though high-level research should be conducted before establishing this as part of the standard treatment for this injury.

When examining the sports outcomes, it is noteworthy that a majority of patients reported in the literature is active in competitive or professional sports.^2,5,7,9-11^ Although this could be due to publication bias, it can also be explained by the repetitive nature of navicular stress fractures, and possibly osteochondral lesions, encountered by high-level athletes. When considering the return-to-sport outcomes in this study and the literature, it should be noted that due to the scant data no reliable return-to-sport estimates can be made. The rehabilitation of professional athletes is highly individualistic and may be subject to a high degree of variation, also including concomitant injuries such as navicular stress fractures or mid-foot injuries. It is important to point out the influence of mental health factors on RTS in this population, as the prolonged nature of recovery and time off play may be important issues driving mental health outcomes.^22^ These considerations should be incorporated in the patient-centered treatment plan.

### Radiological Characteristics

The radiological examination and presentation of TNJ osteochondral lesions has been described in the available literature.^3,4,6,9,10^ By and large, the localization is reported in the central aspect of the navicular bone,^3,4,6,9,10^ which has also been dubbed as the “watershed” area due to its poor blood supply.^19^ One study also reported a single case of a lesion located on the lateral area of the navicular.^4^ In the present study, medial, lateral, and central lesions were observed, though the majority of lesions were located centrally. The lesion morphology described in the literature varies in cysts, fragments, and craters, which concurs with the findings in this study.^3,4,6,9,10^ When considering lesion size, only the imaging study by Bui-Mansfield *et al.*^3^ described their mean dimension (14 mm × 13 mm × 3 mm) from 4 cases. Imaging findings were primarily reported from CT or MRI examinations. Further large database studies are needed to better understand the radiological presentation of these lesions.

### Surgical Technique

The present study described 2 surgical approaches for treating TNJ osteochondral lesions, with an open or arthroscopic approach. The authors consider the choice for a specific approach should be based on the patient characteristics and surgeon preference/experience in an individual basis. A risk-and-benefit analysis should take into account the level of reachability/visibility of the lesion, risk for complications (also by means of vascular status, smoking status, etc.), and patient preference. Previous anatomical and surgical technique studies have shown that both procedures are feasible and safe, with specific guidance for arthroscopic portal placement available.^9,11,23,24^ For both approaches, the authors find that the use of an invasive distractor, such as the Hintermann spreader, is a critical step in achieving adequate working space for a successful treatment.

### Limitations and Future Research

It is important to note that this study is not without limitations. Foremost is its cross-sectional design and low number of patients available. This precludes the authors from statements on the external validity of our findings and did not make any statistical analysis possible. The authors wish to note, however, that TNJ osteochondral lesions are extremely rare, their treatment is challenging, and that the present case series is the largest to date. We therefore hope that the present case series may be of clinical benefit for practitioners encountering these injuries. Another important point to mention is that we did not include any solely conservatively treated patients in our study, although we consider conservative management is an important first step in the treatment of foot and ankle cartilage injuries and should always be considered. Additionally, future studies should focus on measuring treatment outcomes in both operatively and non-operatively treated patients in a prospective manner with ideally a higher number of patients available.

## Conclusion

Surgical treatment of TNJ osteochondral lesions is possible and may lead to successful clinical, sport, and work outcomes. Both open and arthroscopic surgical treatments are available and can be considered in an individual basis based on patient and characteristic of the lesion. This study provides both the description of open and arthroscopic approaches for the treatment of TNJ osteochondral lesions, as well as providing clinicians useful information in the prognosis of this rare clinical entity.
